# Evidence for foot orthoses for adults with flatfoot: a systematic review

**DOI:** 10.1186/s13047-021-00499-z

**Published:** 2021-11-29

**Authors:** Minettchen Herchenröder, Denise Wilfling, Jost Steinhäuser

**Affiliations:** Institute of Family Medicine, University of Lübeck, Ratzeburger Allee 160, 23562 Lübeck, Germany

**Keywords:** Foot orthoses, Shoe inserts, Orthotic devices, Orthotic insoles, Pes planus, Flatfoot, *adults

## Abstract

**Background:**

Flatfoot is characterised by the falling of the medial longitudinal arch, eversion of the hindfoot and abduction of the loaded forefoot. Furthermore, flatfoot leads to a variety of musculoskeletal symptoms in the lower extremity, such as knee or hip pain. The standard conservative treatment for flatfoot deformity is exercise therapy or treatment with foot orthoses. Foot orthoses are prescribed for various foot complaints. However, the evidence for the provision of foot orthoses is inconsistent. The aim of this systematic review is to synthesize the evidence of foot orthoses for adults with flatfoot.

**Methods:**

A computerized search was conducted in August 2021, using the databases PubMed, Scopus, Pedro, Cochrane Library, and the Cochrane Central Register of Controlled Trials. Intervention studies of any design investigating the effects of foot orthoses were included, apart from case studies. Two independent reviewers assessed all search results to identify eligible studies and to assess their methodological quality.

**Results:**

A total of 110 studies were identified through the database search. 12 studies met the inclusion criteria and were included in the review. These studies investigated prefabricated and custom-made foot orthoses, evaluating stance and plantar pressure during gait. The sample sizes of the identified studies ranged from 8 to 80. In most of the studies, the methodological quality was low and a lack of information was frequently detected.

**Conclusion:**

There is a lack of evidence on the effect of foot orthoses for flatfoot in adults. This review illustrates the importance of conducting randomized controlled trials and the comprehensive development of guidelines for the prescription of foot orthoses. Given the weak evidence available, the common prescription of foot orthoses is somewhat surprising.

**Supplementary Information:**

The online version contains supplementary material available at 10.1186/s13047-021-00499-z.

## Background

Foot orthoses are prescribed for various foot complaints and pain [1–3]. Foot orthoses are a common prescription for flatfoot. Flatfoot is characterised by the falling of the medial longitudinal arch, eversion of the hindfoot and abduction of the loaded forefoot. Flatfeet may affect one or both feet [4, 5]. Typical flatfoot symptoms include plantar fascia pain and Achilles tendonitis [6, 7], ligamentous instability and laxity [6], pain under weight loading, rapid fatigability and medial instability in the foot [8]. Furthermore, flatfoot can lead to a variety of musculoskeletal aches in the lower extremity, such as knee pain and hip pain [9, 10]. In the United States five million Americans are currently diagnosed and living with flatfoot [11]. In the UK, the prevalence is estimated to be over 3% in women over 40 years old [12, 13]. Furthermore, 10% of the geriatric population suffers from severe acquired flatfoot due to the degeneration of muscle mass and bone structure [14]. The standard conservative treatment for flatfoot deformity is exercise therapy or treatment with foot orthoses [15]. However, the effect of these varies and remains controversial [16–20].

In Germany, about 8% (five million people) of patients with flatfoot symptoms get a foot orthoses prescription due to any indication. Consequently, there were increased costs in the year 2019 of 466.6 million euros for Statutory Health Insurance [21].

Foot orthoses are mainly prescribed by primary doctors and orthopaedic doctors. In addition, about 30% of primary doctors are general internal medicine physicians in Germany. These two groups have undergone different postgraduate training, e.g. internists are primarily inpatient-based with no surgical or orthopaedic training [22].

It seems like that there are currently no guidelines or checklists for the prescription of foot orthoses for flatfoot, other than those identified in one Delphi consensus study from Australia [23]. Accordingly, the prescription of foot orthoses for flatfoot is often inconsistent and ranges from a purely clinical based approach, to digital motion analysis [24, 25] and force plates [8, 26, 27], to radiological examinations [28–33] or three-dimensional (3-D) imaging [34].

This study aims to systematically review and synthesize the current evidence of foot orthoses for flatfoot.

## Methods

Established methodological frameworks for systematic evidence syntheses [35] and the Preferred Reporting Items for Systematic Reviews and Meta-analyses (PRISMA) [36] were used in order to present results in a full and transparent way and to minimise bias.

No study protocol was registered.

### Search methodology

The search strategy was defined by the principles of a systematic search, using the PICO scheme and implied free-text keywords and medical subject headings (Mesh terms) performed by two reviewers. A computerized search was conducted in August 2021, using the databases Medline via PubMed, Scopus, PEDro, Cochrane Library and Cochrane Central Register of Controlled Trials. Major search terms for all databases are represented in Table S1.

Relevant gray literature was derived via Google Scholar. Furthermore, we checked reference lists of included studies, and relevant reviews were identified through the search. The results of the search were imported into the web service Covidence (www.covidence.org), which was used for the entire review process.

### Study selection

All intervention studies evaluating any kind of foot orthoses or inserts for flatfoot were eligible for inclusion, with the exception of case studies. Studies evaluating surgical interventions were excluded. We included scientific articles published in peer reviewed journals in English and German. To get the largest possible number of studies, no limitations of the publication year were applied. As an inclusion criterion, studies must have determined outcomes in the form of patient-reported, clinician-reported or laboratory-based measurements. All inclusion and exclusion criteria are shown in Table 1.

**Table 1:** Inclusion and exclusion criteria

Inclusion	Exclusion
English-language articles German-language articles	Articles in other languages
Scientific articles published in peer-reviewed journals	Popular articles Study summaries
All kind of intervention studies	Case reports Systematic reviews
All kid of shoe insoles	Other interventions
Adult patients with flatfoot	children
Outcomes measured with any kind of tool	Outcomes not measured with any tool
All settings	

Two independent reviewers (MH, DW) assessed titles and abstracts from all search results to identify eligible studies. After potentially relevant articles were selected, full reports were obtained and inclusion and exclusion criteria were assessed. Intervention studies evaluating any kind of foot orthoses for adults with flatfoot were included. Studies evaluating surgical interventions were excluded.

Any disagreement on the eligibility of studies was resolved through discussion to reach consensus or, if required, by involving a third experienced review author (JS).

### Data extraction and methodological quality

Data from each study included in this review was independently extracted by two reviewers (MH, DW). In the event of disagreements or discrepancies, a third review author was involved to reach consensus. Data were extracted into standardised tables, including author, publication year, study design, participants, interventions, setting, outcomes, measurements and main findings according to the Cochrane Handbook for Systematic Reviews of Interventions [35].

The methodological quality of all of the included studies was assessed using the Cochrane Risk of Bias tool for randomized controlled trials (RCTs) [35] and the ROBINS-I tool for all other study designs. Two reviewers (MH, DW) independently assessed the methodological quality of the studies included in order to identify any potential sources of bias.

## Results

### Description of included studies

**Fig. 1:**
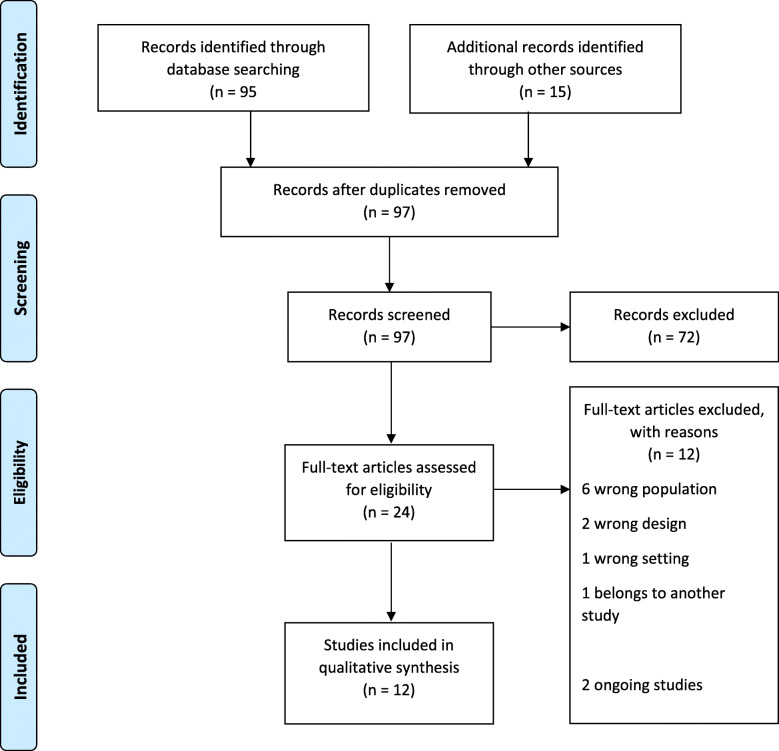
PRISMA Flow Chart

The initial search identified 110 studies. After removing duplicates, 97 studies were investigated. 72 studies were excluded after title and abstract screening. 24 full texts were reviewed and finally 12 studies were included in the study. Two ongoing studies were also included [37, 38]. The process of study selection is presented in Fig. 1.

A total of 170 participants were included. The sample sizes of the included studies ranged from 8 [34] to 80 [39]. Of the 12 included studies, most were repeated-measures intervention studies (*n* = 9) [16, 34, 40–46]. Other studies used a randomised-controlled design [39], before-after design [47] or a non- randomised controlled design [48].

Studies were conducted in Asia [16, 34, 39, 43–46, 48], Australia [42], the United States [41], Iran [40] and Turkey [47]. Most participants were young adults aged between 18 and 45 years (Mean = 31.5) [16, 34, 41, 42, 46–48]. Two studies recruited only male participants aged 20 and 22 years (Mean = 21) [40, 45] and three studies recruited only college students [43–45]. Two studies [43, 44] gave no information about age.

Flatfoot was diagnosed differently in the included studies. Chen et al. [16] diagnosed flatfoot by static foot posture, Kido et al. [34] based their diagnosis on radiographs under static loading conditions and Murley et al. [42] investigated their participants radiologically and applied an arch index. Tang et al. [48] diagnosed flatfoot by measuring the arch index described by Cavanagh and Rodgers [49]. Xu et al. [39] diagnosed flatfoot by foot posture index (FPI). Other studies gave no information about flatfoot diagnosis [40, 41, 43–47].

Only one study [47] described the study setting. None of the studies provided information on wearing time or when the outcomes were measured. Study characteristics are summarized in Table 2.

**Table 2:** Characteristics of included studies

Author/year/country	Design	Population	Intervention	Setting	Outcomes/Measurement	Main results
Acak 2020 (Turkey)	Before-after study	34 participants (17 male) with pes planus complaints Age: 18–28 years	Individually designed insoles: 1 mm thick stainless chrome steel covered with 3 mm thick antibacterial leather	Orthopedics and Traumatology Department of Turgut Ozal Medical Center in Inonu University	Outcomes: Height, weight, percent body fat, 30 m sprint test, vertical jump, 12 min Cooper test and Visual Analog Scale (VAS) Measurement: Image of the soles of feet were obtained by using the podoscope device.	Statistically significant differences found in pre- and post-test results in weight, BMI, 30 m run, vertical jump, 12 min Cooper run and VAS
Aminian et al. 2013 (Iran)	Repeated- measures intervention studies	12 participants (12 male) with flexible flatfoot Age: 22.25 (±1.54)	Prefabricated orthosis: commercially available, full length and made of two layers: ethyl vinyl acetate at the bottom layer and 1 mm thick leather layer on the top. Proprioceptive orthosis: made of rubber and covered by cloth, 2 mm thick insole with no arch support; 3 mm wedge as an elevation area extending from the navicular to the hallux and slopped medial to lateral.	No information	Outcomes: In-shoe plantar pressure (medial heel, lateral heel, medial midfoot, lateral midfoot, first ray, second and third rays, fourth and fifth rays) Measurement: Pedar-X system under 3 conditions (wearing the shoe only, wearing the shoe with prefabricated insole, wearing the shoe with proprioceptive insole)	Proprioceptive insoles: maximum force was significantly reduced in medial midfoot, and plantar pressure was significantly increased in the second and third rays compared to the shoe only condition. Prefabricated insole: maximum force was significantly higher in midfoot area compared to the other conditions
Chen et al. 2010 (Taiwan)	Repeated- measures intervention studies	11 participants (6 male) with flatfoot Age: 45.9 (±15.66)	Insoles: custom made of vinyl-acetate and 12 ± 3% far-infrared nanopowders Shoes: custom made of rubber and PU	No information	Outcomes: Spatio-temporal parameters, kinematic and kinetic data Measurement: eight-camera Eagle digital motion analysis system, using 15 spherical retro-reflective markers under three test conditions: walking barefoot, walking with shoes, and walking with shoes and insoles,	Walking with shoes and insoles and walking with shoes: increased the peak ankle dorsiflexion angle and moment, reduced the peak ankle plantarflexion angle and moment, increased the peak knee varus moment. Effects of the orthoses on knee and hip were minimal and no significant differences were observed between walking with shoes and insoles and walking with shoes.
Han et al. 2019 (South Korea)	Repeated- measures intervention studies	28 participants (male college students) with flatfoot Age: 20.29 (±0.46) Weight: 70.43 (±4.15) kg Height: 1.75 (±3.55) cm	Three different insoles: The normal insoles were used as an experimental control without arch support function Type A insole With only arch support function Type B insole With both arch support and cushion pads for shock absorbing functions Type A and B Hardness and foot arch descent 45°	No information	Outcomes: Compute the range and peek of Rearfoot motion (RFM) and ankle joint Measurement: 10 Vicon Motion Capture System was used. 21 reflective markers were attached with three different insoles	Insoles A and B show significantly less rearfoot ankle movement than the normal insole.
Jiang et al. 2021 (China)	Repeated- measures intervention studies	10 participations (8 male, 2 females Age: 30 years with flexible flatfoot	Three different insoles: Type A: the insole was obtained by 3D printing from the plantar pressure (PPRI) Type B: Orthotic insole Type C Flat insole	No Information	Outcomes Plantar pressure stance time, stride frequency and peak pressure in each area of the sole and plantar condistribution Measurement Walking on treadmill at low, normal and fast speed with the different insoles	Force on the hindfoot and midfoot increased significantly when wearing flat insoles compared to PPRI and orthopedic insoles. Contact area at slow and normal speed in the midfoot area is smaller when wearing PPRI than with flat insole
Kido et al. 2014 (Japan)	Repeated- measures intervention studies	8 participants (4 male, Age 29–38; 4 females, Age 26–38) with mild flatfoot deformity	Accessory insoles Therapeutic insoles: deformity: made using a CAD system (Pedcad Insole Designer; Pedcad, Oberkochen, Germany), designed to raise the medial longitudinal arch by 10 mm with a 5 mm inner wedge, particularly reducing the burden of the posterior tibial tendon	No information	Outcomes: tibia and the tarsal and metatarsal bones of the medial longitudinal arch (i.e., first metatarsal bone, cuneiforms, navicular, talus, and calcaneus) Measurement: Three-dimensional CT models; tibia and the tarsal and metatarsal bones of the medial longitudinal arch (i.e., first metatarsal bone, cuneiforms, navicular, talus, and calcaneus)	Therapeutic insoles: significantly suppressed the eversion of the talocalcaneal joint The subjects voiced no complaints of discomfort
Miller et al. 1996 (United States of America)	Repeated- measures intervention studies	25 participants (13 male,12women) with asymptomatic pes planus Age: 18–40 year	Orthotic device: constructed by using a plastic polymer and a firm Plastizote medial heel wedge	No information	Outcomes: the dynamic GRFs (ground reaction forces) as a percentage of body weight in three directions-vertical, mediolateral, and anteroposterior-and the center of pressure by using an xand y-axis. Measurement**:** Walking across a standard force plate in 10 trials with and 10 trials without an orthotic device	Orthotic device: reduces vertical and anteroposterior GRFs in the early stages of the stance phase during the gait cycle. No evidence was found to conclude that either the center of pressure or the mediolateral GRF showed any significant change when a standard street shoe was used with and without an orthotic device.
Murley et al. 2010 (Australia)	Repeated- measures intervention studies	30 subjects (15 male) with flatfeet Age: 18–37 years	Customized FO: a plaster cast impression was taken of each participant’s feet, made from a semi-rigid 4 mm polypropylene thermoplastic shell and included features considered to minimize rearfoot pronation Modified prefabricated FO: three-quarter-length Formthotic made from dual-density polyethylene foam	No information	Outcomes: Comfort rating, electromyographic activity, foot posture Measurement: VAS Scale **(**baseline and after 12 days), Electromyogram, X-rays, under 4 conditions: Four experimental conditions were assessed: (i) barefoot, (ii) shoe only, (iii) a heat-moulded (modified) prefabricated foot orthosis, and (iv) a 20-degree inverted-style customized foot orthosis.	Results show significant changes in EMG amplitudes of the tibialis anterior with both FOs, but only the prefabricated FO had a significant effect on EMG
Park et al. 2017 (Republic of Korea)	Repeated- measures intervention studies	15 participants (college students) with flatfoot	Functional foot orthotic (FFO): customized for each individual’s foot shape and created with thermoplastic materials, high-density resistance elastic pad, cup sole for the plantar arch, low-elasticity pad for shock absorption in the heel, and ethylenevinylacetate (EVA)	No information	Outcomes: change in the pelvic angle Measurement: six MX-F40 cameras, two OR6–7 force plates; walking on a previously fabricated Walkway before and after wearing the customized FFOs	Large changes in the pelvic angle on both the left and right sides during the pre-stance and mid-stance and pre-swing and midswing periods of the gait cycle before wearing the orthotic. These changes decreased significantly after wearing the orthotic (*p* = 0.05)
Peng et al. 2020 (China)	Repeated- measures intervention studies	15 participants (9 male) with flatfoot	Prefabricated insoles: 3 cm thick medial arch support and 6 inclined medial forefoot posting, made of fabric with embedded cushioning silicon at the heel region Running shoes (Reebok Run Supreme 4.0)	No information	Outcomes: hip, patellofemoral, ankle, medial and lateral tibiofemoral joint contact forces Measurement**:** 3D-motion capture system, 4 force plates under two conditions: walking with shoes and foot orthoses and walking with shoes	Prefabricated insoles: second peak patellofemoral contact force and the peak ankle contact force were significantly lower, significantly reduced the peak ankle eversion angle and ankle eversion moment, the peak knee adduction moment increased
Tang et al. 2015 (Taiwan)	Controlled- trial	Intervention Group: 10 subjects (age 15–45) with flexible flatfoot Control Group: 15 subjects (age-matched) without flatfoot	Total contact insole: Custom-made, total foot contact with extended heel guard to keep subtalar joints in neutral position, forefoot medial posting, double-layer composition with superficial PPT and semi rigid plastozote base	No information	Outcomes: rearfoot motion and plantar pressure redistribution Measurement: motion analysis system under three test conditions (walk with barefoot, walk with sports shoes, and walk with TCIFMP and sports shoes)	Total contact insole: tends to reduce valgus angle and becomes statically similar to normal subjects, reduced foot pressure in the hallux and heel area compared to those wearing only sports shoe.
Xu et al. 2019 (China)	Randomized-controlled- trial	Intervention Group: 40 subjects (20 males, 20 females) with flexible flatfoot Age: 26–55 Weight: 63.37 (± 12.52) kg Control Group: 40 subjects (20 males, 20 females) with flexible flatfoot Age: 26–60 years Weight: 67.18 (± 10.72) kg	Individually designed insoles 3 D print insoles with standardize shoes Customized insoles Standardize shoes with customized ethylene vinyl acetate (EVA) insoles	Norman Bethune Second Hospital of Jilin University	Outcomes: VAS was measured to measure comfort at 0 and 8 weeks Measurement: Footscan was used to measure plantar pressure under three test conditions: barefoot, with 3 D insole and standardized insole. 3 walking trials over a 10 m walking distance, at a speed of 3.12 (± 1.95) km/h. The insoles were worn every day for 6–8 h over 8 weeks.	At week 0, peak pressures in the midfoot were significantly lower (*p* < 0.05) in the experimental group compared to the control group At week 8, peak pressures in the midfoot were significantly higher (p = 0.05) in the experimental group compared to the control group Comfort scores (measured anhnad by VAS) were significantly (*p* = 0.05) lower in the experimental group than in the control group

### Methodological quality

For the randomised-controlled study of Xu et al. 2019, we judged the risk of bias as low in all items according to the Cochrane risk of bias tool for randomised controlled trials [50]. We were unable to judge the overall risk of bias for the other included studies because of a lack of information in the study reports. There is no clear indication that the studies are at serious or critical risk of bias and there was a lack of information in one or more key domains of bias [51]. None of the included studies [16, 34, 40–44, 46–48] gave information about confounding, selection of participants, deviations from intended interventions as well as selection of reported results. In only two studies [47, 48] the risk of bias due to missing data was determined to be low because outcome data was reported for all study participants. In other studies, [16, 34, 40–44, 46] it was unclear if outcome data included data from all participants. The risk of bias in classification of intervention was judged to be low in all studies [16, 34, 40–44, 46–48] because intervention groups were clearly defined. All studies [16, 34, 40–44, 46–48] showed a low risk of bias in measurement of outcomes because studies used objective outcome measurements. Results of the risk of bias assessment are summarized in Table 3.

**Table 3:** Risk of bias judgments by ROBINS-I domains

	ROBINS-I domains								
Author	Year	Bias due to confounding	Bias in selection of participants	Bias in classification of intervention	Bias due to deviations from intended interventions	Bias due to missing data	Bias in measurement of outcomes	Bias in the selection of reported results	Overall
Acak et al.	2020	NI	NI	Low risk	NI	Low risk	Low risk	NI	NI
Aminian et al.	2013	NI	NI	Low risk	NI	NI	Low risk	NI	NI
Chen et al.	2010	NI	NI	Low risk	NI	NI	Low risk	NI	NI
Han et al.	2019	NI	NI	Low risk	NI	NI	Low risk	NI	NI
Jiang et al.	2021	NI	NI	Low risk	NI	NI	Low risk	NI	NI
Kido et al.	2014	NI	NI	Low risk	NI	NI	Low risk	NI	NI
Miller et al.	1996	NI	NI	Low risk	NI	NI	Low risk	NI	NI
Murley et al.	2010	NI	NI	Low risk	NI	NI	Low risk	NI	NI
Park et al.	2017	NI	NI	Low risk	NI	NI	Low risk	NI	NI
Peng et al.	2020	NI	NI	Low risk	NI	NI	Low risk	NI	NI
Tang et al.	2015	NI	NI	Low risk	NI	Low risk	Low risk	NI	NI

NI: no information

### Measurements

Most studies [16, 34, 47, 48] measured the effect of foot orthoses via 3-D motion capture. Chen et al. [16] used eight cameras under three conditions: walking barefoot, walking with shoes only and walking with shoes and foot orthoses. A motion analysis system was used by Tang et al. [48] to measure the effect of foot orthoses under the same conditions as Chen et al. Kido et al. [16, 34] used computed tomography. Acak [47] used a podoscope device to make images of the soles of feet. Han et al. [45] used 10 cameras under three conditions: walking with normal foot orthoses, foot orthoses with only arch support function and foot orthoses with both arch support and cushion pads for shock absorbing functions.

Pressure measurement plates were used in four studies [39–41, 46]. Miller et al. [41] conducted ten trials with and without foot orthoses. Aminian et al. [40] measured three conditions: wearing the shoe only, wearing the shoe with prefabricated foot orthoses and wearing the shoe with proprioceptive foot orthoses. Xu et al. [39] measured three conditions: barefoot, with 3-D and standardized foot orthoses. Jiang et al. [46] measured three different conditions: using an orthotic insole, a flat foot insole and 3-D printed insole. Two studies [43, 44], used imaging as well as pressure plates. In Murley et al. [42], imaging and electromyogram were conducted at baseline. After 12 days, four conditions were investigated: barefoot, walking with shoe only, walking with prefabricated foot orthoses and with 20 degree inverted customized foot orthoses.

### Outcome measures

All included studies used different outcome measures. None of the studies reported an adverse event.

### Plantar pressure assessment

Aminian et al. [40] measured plantar pressure in the shoe at the medial and lateral heel, medial and lateral midfoot and first, second, third, fourth and fifth rays. For the proprioceptive orthosis, the maximum force was significantly reduced in the medial midfoot, and plantar pressure was significantly increased in the second and third rays compared to the shoe only condition. Similarly, Jiang et al. [46] measured plantar pressure during walking at slow, normal and fast gait speeds on the treadmill at the hindfoot and midfoot while wearing 3-D foot orthoses (PPRI), foot orthoses and flat foot orthoses. The force on the rear and midfoot was significantly increased when wearing flat foot orthoses compared to PPRI and foot orthoses. In addition, the contact area at slow and normal speed in the midfoot area was smaller with PPRI compared to flat foot orthoses. In contrast Park et al. [43] investigated the change in pelvic angle using foot orthoses and reported a significant decrease after wearing the orthotic. Peng et al. [44] investigated the patellofemoral joint and the medial and lateral tibiofemoral joint. After wearing the foot orthoses, the second peak patellofemoral contact force and the peak contact force of the ankle were significantly lower. Foot orthoses also significantly reduced the peak eversion angle and the eversion moment of the ankle. The peak adduction moment of the knee was increased. Rearfoot motion and plantar pressure redistribution were measured in Tang et al. [48]. They reported that foot orthoses may reduce valgus angle and becomes statically similar to normal participants. Furthermore, a reduced foot pressure in the hallux and heel area when wearing foot orthoses was found compared to those wearing only sports shoes. Han et al. [40] reported that hindfoot ankle motion was less with both custom-made foot orthoses than with the normal foot orthoses. Xu et al. [39] measured three conditions: barefoot, with 3-D and standardized foot orthoses.

### 30 m jump test, vertical jump test and 12 min Cooper-test

Acak [47] measured BMI and weight and conducted a 30 m jump test, a vertical jump test, the 12 min Cooper-test and Visual Analog Scale VAS. For the individually designed foot orthoses, they found statistically significant differences in all mentioned categories between pre- and post-test.

### Motion analysis

Chen et al. [16] investigates spatio-temporal parameters as well as kinematic and kinetic data. For the custom-made foot orthoses, the following was found: an increase in the peak ankle dorsiflexion angle and moment, a reduction in the peak ankle plantarflexion angle and moment as well as an increase in the peak knee varus moment. Kido et al. [34] examines tibia, tarsal and metatarsal bones and medial longitudinal arch. The therapeutic foot orthoses significantly suppressed the eversion of the talocalcaneal joint.

### Dynamic ground reaction forces

Miller [41] measured dynamic ground reaction forces (GRFs) in three directions: vertical, mediolateral and anteroposterior. The orthotic device reduces vertical and anteroposterior GRFs in the early stages of the stance phase during gait cycle.

### Electromyography

In the study conducted by Murley et al. [42] electromyographic activity and foot posture was measured and the prefabricated foot orthosis showed a significant effect.

## Discussion

The aim of this review was to summarize the evidence of foot orthoses in adults with flatfoot. Altogether, twelve studies could be included in this review. However, only one of these was a randomised controlled trial. Studies investigated different foot orthoses, such as custom-made foot orthoses [16, 39, 42–48], uniformly manufactured foot orthoses [34, 40] and semi-rigid foot orthoses [41]. None of the studies gave information about the selection of foot orthoses for the treatment of flatfoot. Additionally, authors used inconsistent terminology for the terms ‘orthotic device’ and ‘foot orthoses’. For example, some studies used the term orthotic device [40, 41, 43] when referring to foot orthoses.

Studies used different methods for the diagnosis of flatfoot in participants. One reason for this could be the lack of a standardized framework for flatfoot diagnosis [52–54].

A guideline should give advice for the selection of foot orthoses [52, 55]. Compared to our study, similar results were found in reviews of the evidence for non-surgical interventions for pediatric flatfoot [52, 55]. In this context, it should be mentioned that in Australia there is a guideline from the year 1998 for the prescription of foot orthoses from Petchell et al. [56] and from the year 2014 from Banwell et al. [23] that regulates the prescription of custom-made insoles for adult flatfoot. In Germany, there is currently no guideline for the provision of foot orthoses for adults with flatfoot.

The studies included provided little information in relation to how participants were recruited. Furthermore, no information was given on the severity of participants’ symptoms. The majority of participants may have felt healthy and had no pain or other complaints. Banwell et al. [54] stated that it would be more appropriate to investigate the effectiveness of foot orthoses within a population recognizing symptoms.

The studies included assessed different outcomes and due to inconsistent outcome measurement, results are difficult to interpret. It is noteworthy that only two studies [34, 39] assessed the comfort of wearing the foot orthoses. The user’s comfort should certainly be considered, as discomfort may be an important factor influencing patient’s adherence. Furthermore, most of the included studies lack information on wearing time or when the measurements were conducted. Consequently, the comparability is low. Only one study reported on the observation period [42].

Although all studies found positive impacts, results must be interpreted with caution. We only found one randomised controlled trial evaluating the effectiveness of foot orthoses for patients with flatfoot. Most of the studies (*n* = 9) were repeated-measures intervention studies. In this design, participants are measured two or more times on the same variable, are given different treatments and measurements are taken after each one. This means that each participant will act as their own control [57, 58].

Due to a lack of information in several studies, no conclusive statement can be made about risk of bias. The studies which evaluate the effect of foot orthoses are predominantly repeated-measures intervention studies. The low number of randomised controlled trials leads to a lack of evidence. This result is consistent with the findings of Banwell et al. [54], who also found predominantly repeated-measure intervention studies. Additionally, the reporting quality of studies is rather low and important information about study methodology is missing.

Two [16, 41] studies out of twelve provided no information about any conflicts of interest. Two studies [34, 42] received funding but did not declare any conflict of interest.

Other reviews [55, 59] also reported that there is no strong evidence regarding the effect of foot orthoses.

The results of this review are consistent with the results of other reviews [52, 54, 59] addressing the evidence for foot orthoses. Based on the available evidence and the absence of clinical guidelines, the routine prescription of foot orthoses for flat feet in adults needs to be reconsidered [23, 60]. Unfortunately, we are unable to draw firm conclusions due to the lack of methodological and reporting quality of the included studies. Furthermore, randomised trials with foot orthoses under standardized conditions need to be conducted, as these are the gold standard in testing evidence for efficacy [61].

To minimise bias, the whole screening and data extraction process was conducted by two independent researchers. Furthermore, we contacted study authors and experts in the field for unpublished studies.

A limitation of this review is that only a small number of studies were suitable for inclusion and the studies included almost all used a repeated-measure intervention design. Because there is no separate tool to assess methodological quality of repeated-measure intervention studies, the ROBINS-I tool was used. The methodological quality of all of the included studies was determined to be unclear due to the insufficient quality of reporting. A further limitation of this review is as follows: due to the lack of randomised-controlled trials, the potential for confounding and bias could not be ruled out. A comparison of the studies was not possible because all of the included studies measured different outcomes, using different measurement methods.

Because of this number of limitations, no clear conclusion regarding effectiveness and effects of foot orthoses can be drawn and it is not possible to make a clear recommendation for the use of foot orthoses in adults.

## Conclusions

As appropriate studies were not detected in our searches, no firm conclusions can be drawn on the effects and effectiveness of foot orthoses for patients with flatfoot. However, there are several limitations, such as the inconsistent population of participants studies, the various foot orthoses used, the different outcomes and the fact that no temporal information about the wearing of the foot orthoses was described. Evidence in terms of diagnostics, indication and the prescription of different kinds of foot orthoses, as well as evidence concerning duration of therapy, is needed. To date, no firm conclusions can be drawn about the (positive) effects foot orthoses might have for patients with flatfoot. Against this background, the common prescription of foot orthoses in adults with flatfoot is surprising.

## Supplementary Information

**Additional file 1.** Table S 1: Search terms to select the studies

## Data Availability

Not applicable.
