# Supplementary material for: Evidence for foot orthoses for adults with flatfoot: a systematic review
Source: J Foot Ankle Res. 2021 Nov 29;14:57. doi: 10.1186/s13047-021-00499-z (PMC8628393; doi:10.1186/s13047-021-00499-z)
Supplement: Supplementary file 1 — Additional file 1. Table S 1: Search terms to select the studies [file 13047_2021_499_MOESM1_ESM.docx]

Table S 1: Search terms to select the studies

|  |  | shoe insoles *OR* |  |  |
| --- | --- | --- | --- | --- |
| flatfoot [MeSH] *OR* |  | shoe inserts *OR* |  | children |
| pes planus *OR* | **AND** | shoe orthotics *OR* | **NOT** | pediatric |
| valgoplanus |  | arch insoles *OR* |  |  |
|  |  | non-surgical* |  |  |
